# Nomogram for prediction of non-proliferative diabetic retinopathy in juvenile-onset type 1 diabetes: a cohort study in an Asian population

**DOI:** 10.1038/s41598-018-30521-7

**Published:** 2018-08-15

**Authors:** Eugene Yu-Chuan Kang, Fu-Sung Lo, Jung-Pan Wang, Lung-Kun Yeh, An-Lun Wu, Yun-Ju Tseng, Chun-Ting Yeh, Laura Liu, Kuan-Jen Chen, Wei-Chi Wu, Chi-Chun Lai, Nan-Kai Wang, Chang Gung Juvenile Diabetes Eye Study Group, Tun-Lu Chen, An-Ning Chao, Yih-Shiou Hwang, Yen-Po Chen, Yih-Hsin Chen

**Affiliations:** 1 Department of Ophthalmology, Linkou Medical Center, Chang Gung Memorial Hospital, Taoyuan, 333 Taiwan; 2 Chang Gung University, College of Medicine, Taoyuan, 333 Taiwan; 3 Division of Pediatric Endocrinology and Genetics, Linkou Medical Center, Chang Gung Memorial Hospital, Taoyuan, 333 Taiwan; 4 Department of Orthopaedics & Traumatology, Taipei Veterans General Hospital, Taipei, 112 Taiwan; 5 School of Medicine, National Yang-Ming University, Taipei, 112 Taiwan; 6 Herbert Irving Comprehensive Cancer Center, Columbia University, New York, NY 10032 United States; 7 Chang Gung University, School of Traditional Chinese Medicine, Taoyuan, 333 Taiwan; 8 Department of Ophthalmology, Edward S. Harkness Eye Institute, Columbia University, New York, NY 10032 United States

## Abstract

The need for screening for retinopathy in patients with type 1 diabetes mellitus (T1DM) has been emphasised, but diagnostic delays were reported when screening was done at fixed intervals. To establish an individualised risk-prediction model to assist screening non-proliferative diabetic retinopathy (NPDR) in T1DM, we performed a retrospective cohort study enrolling participants in the Chang Gung Juvenile Diabetes Eye Study. There were 413 patients with 12 381 records analysed from 2005 to 2015. A time-dependent Cox proportional hazard analysis was used to evaluate the risks of NPDR development and a nomogram with risk-stratification indicators was established based on the results. During 97 months of follow-up, 43 of 413 patients (10.4%) developed NPDR. Male sex (HR: 0.4, 95% CI: 0.19–0.85), age 5–14 years at onset of T1DM (6.38, 2.41–16.87), duration of diabetes (1.57, 1.41–1.75), and hemoglobin A1c level (1.56, 1.35–1.80) were independently associated with NPDR. Using the nomogram offers a quick method in the clinical setting to interpret the risk of NPDR development. Based on its weighting, each of the independent factors is allocated a score, and the total points indicate the probabilities of NPDR occurring within 6 months, 1 year, and 3 years.

## Introduction

In recent years, an increasing number of patients have been diagnosed with type 1 diabetes mellitus (T1DM) in many countries^1–3^. A nationwide study conducted on Taiwan reported a significant increase in the number of patients diagnosed annually with T1DM between 1999 and 2010^4^, and raised the awareness of the disease’s morbidity and complications^4–6^. Vascular (including retinopathy, nephropathy, and hypertension) complications are common in juvenile-onset T1DM patients^6^, and treatment for the disease may cost more than $16,000 annually for each patient with complications^7^.

Diabetic retinopathy (DR) is one of the leading microvascular complications of T1DM^5^. It is also one of the primary causes of blindness in working-age adults and is an extremely serious issue^8^. Previous studies emphasised that establishing an appropriate screening policy is mandatory for early detection and treatment of DR in patients with T1DM^6,9^, and reducing the risk of severe vision loss^10^. According to the guidelines of the American Academy of Ophthalmology (AAO)^11^ and the American Diabetes Association (ADA)^12^, annual DR screenings should begin 5 years after T1DM is diagnosed or 3–5 years after the diagnosis if the patient is ≥10 years old. However, a delay in the diagnosis of DR was reported in some patients when screening began 3–5 years after the initial T1DM diagnosis^13^. Recently, the Diabetes Control and Complications Trial/Epidemiology of Diabetes Interventions and Complications (DCCT/EDIC) Research Group used fundus photography to advocate an individualised screening process and established a schedule for T1DM patients based on hemoglobin A1c (HbA1c) instead of a fixed time period^14^. Although that study mainly focused on the progression of retinopathy, it indicated that the risk of DR varied between patients because of differences in glucose control and other variables. Hence, our aim was to develop an individualised prediction model based on the patient’s characteristics to improve the screening policy.

In this retrospective study, we analysed risk factors associated with non-proliferative diabetic retinopathy (NPDR) development in patients with juvenile-onset T1DM by creating a nomogram and establishing a risk-stratification indicator. The nomogram is an economically viable method that can be easily used by pediatricians and ophthalmologists to early predict the risk of NPDR in juvenile-onset T1DM patients.

## Materials and Methods

### Data acquisition and study population

This retrospective cohort study was performed using the database from the Chang Gung Juvenile Diabetes Eye Study (CGJDES), which is a part of the nationwide Diabetes Shared Care Program in Taiwan. Patients may be referred from local practitioners or other specialists for integrated patient care after T1DM has been diagnosed. CGJDES has collected comprehensive medical information from T1DM patients at the Linkou and Taipei branches of Chang Gung Memorial Hospital (CGMH) since 2005. A detailed description of the CGJDES recruitment protocol was previously described^15^. The study was approved by the Ethics Institutional Review Board of CGMH (IRB No. 103–3203B), and the principles of the Declaration of Helsinki were applied. The need for informed consent was waived by the Ethics Institutional Review Board. The study population included all T1DM patients enrolled in CGJDES between 2005 and 2015. The exclusion criteria were (1) the onset of T1DM at age ≥18 years and (2) the first fundus screening disclosing a presence of DR. During the follow-up, the patients underwent an annual laboratory screening, testing of HbA1c every 3–12 months, and annual ophthalmological examinations and fundus photography. Patients were excluded if the data for these parameters were inadequate, i.e. less than 2 for HbA1c and less than 1 for other data or fundus photography, for any year of the study.

### Ophthalmic examination

All patients underwent comprehensive eye examinations annually, which included taking a measurement of visual acuity, a slit-lamp examination, and three-field fundus photography after pupil dilation. The fundus photography was evaluated by retinal specialists and DR severity was classified according to the Early Treatment of Diabetic Retinopathy Study (ETDRS)^16^. The retinal specialists were not given any information about the demographics and other diabetes complications of the patients. The patients were followed and treated under the guidelines of the AAO^11^ and the ADA^12^.

### Demographics and risk factors

Demographic information for the patients was collected, including sex, baseline age, age at onset of T1DM, overall duration of T1DM, and duration of follow-up. Baseline age was defined as the age at the time of enrolment in CGJDES. The baseline body mass index (BMI) was calculated by taking the patient’s body weight in kilograms divided by the square of height in meters. Regular laboratory examinations were performed, and data for serum HbA1c, serum creatinine, and serum lipids were obtained.

### Statistical analysis

The data were summarised using frequency and proportion for categorical variables, and mean and standard deviation for continuous covariates. We performed a time-dependent Cox proportional hazard analysis to evaluate the risk factors for NPDR. The dataset was composed of time-invariant (time-independent) covariates and time-varying (time-dependent) covariates. The time-invariant covariates included sex, BMI and age at onset of T1DM. Time-varying covariates included the duration of T1DM at each HbA1c examination, HbA1c at each examination, and averaged lipid profiles (including low-density lipoprotein [LDL], cholesterol, triglycerides, and serum creatinine) for a given year. The time-dependent analysis updated the survival time when the subsequent HbA1c result was registered, allowing this experiment to be considered similar to the nature of the dynamic process in a real-world environment. The nomogram was designed based on the results from the above analysis. The performance and discriminative ability was assessed using C-statistics (Harrell’s concordance index). The extent of the model’s generalisability was evaluated using an internal validation by comparing the difference in C-statistics between the original optimistic estimate and the bootstrap-corrected estimate. Statistical analyses and nomogram establishment were performed using the R statistical package (version 3.4.0; R Development Core Team) and the ‘rms’ package (Version 5.1–1 updated on May 1, 2017).

## Results

### Patient enrolment

**Figure 1:**
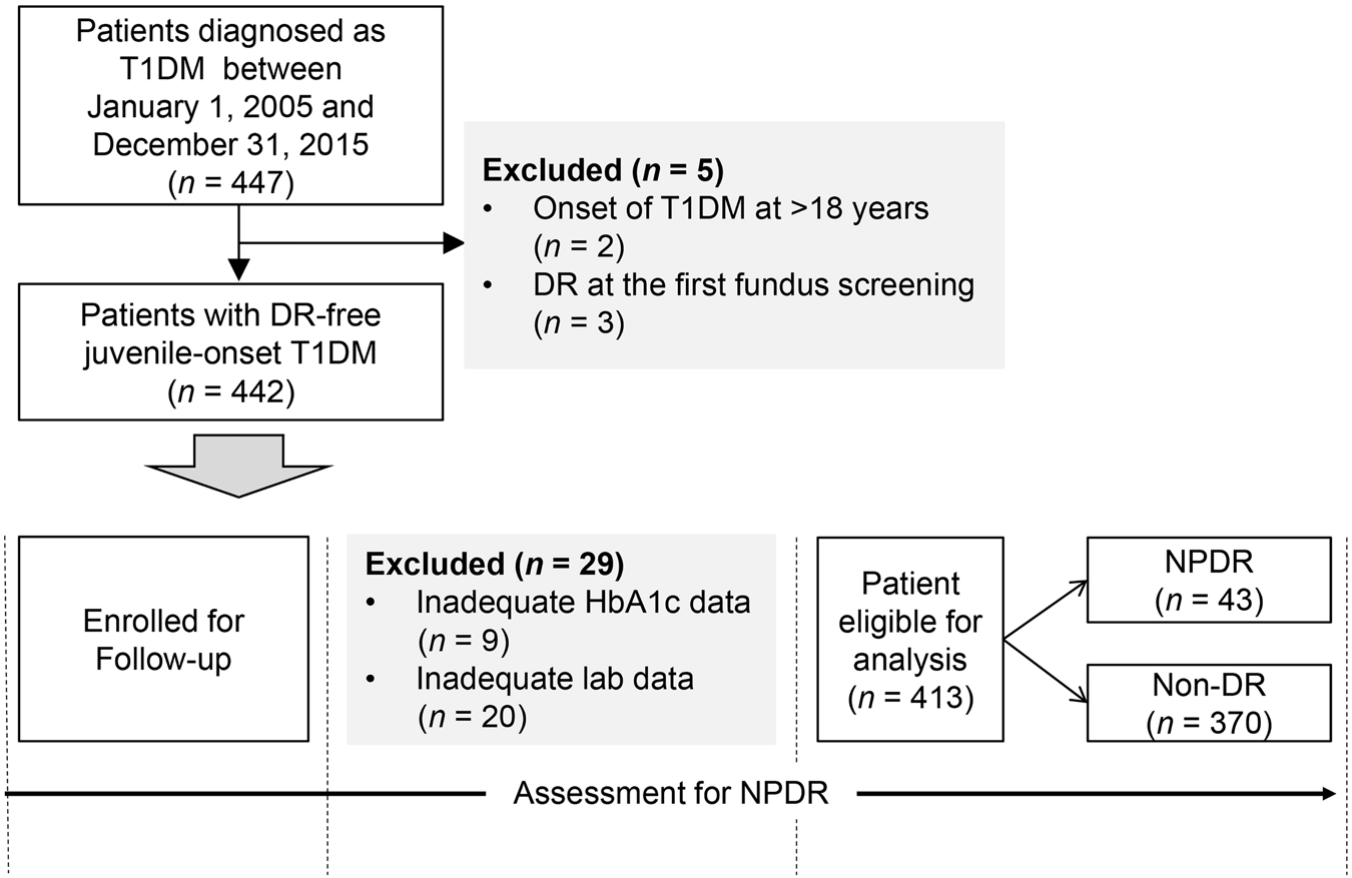
The distribution of study participants. T1DM: type 1 diabetes mellitus, NPDR: non-proliferative diabetic retinopathy, HbA1c: hemoglobin A1c.

From 2005 to 2015, 447 patients diagnosed with T1DM were registered in CGJDES. We excluded two patients with an onset of T1DM at >18 years, three patients who had DR at the first fundus screening, and 29 patients because of inadequate data for HbA1c or other laboratory results (Fig. 1). Of the 413 patients with juvenile-onset T1DM who were eligible for analysis, 43 (10.4%) developed NPDR during the follow-up according to the ETDRS grading^16^. There were 12 381 records for the 413 patients, with the minimum per patient being 2 records and the maximum being 68 records.

### Demographics of the study participants

The demographic characteristics, BMI, and laboratory data were recorded for the study cohort (Table 1). There were 205 (49.6%) male patients in this population. The baseline age at registration in CGJDES was 10.6 years, the age at T1DM diagnosis was 8.4 years, the overall duration of T1DM was 126.3 months, and the duration of follow-up in CGJDES was 97 months. At the first enrolment into CGJDES, patients’ BMI and HbA1c levels were 18.5 kg/m^2^ and 10·0%, respectively. The overall average results of laboratory testing during the follow-up were 99.1 mg/dl for LDL-cholesterol, 172.4 mg/dl for total cholesterol, 74.6 mg/dl for triglycerides, 63.8 mg/dl for high-density lipoprotein (HDL)-cholesterol, and 0.6 mg/dl for serum creatinine. Regarding patients with abnormal baseline laboratory data, 30.8%, 48.2%, 18.9%, 3.4%, and 0.7% of participants had abnormal LDL, total cholesterol, triglyceride, HDL, and creatinine levels, respectively. The mean HbA1c level for the 12 381 records from 413 patients during the follow-up was 8.8 ± 2.1% (data not shown).

**Table 1:** Demographic characteristics of patients with juvenile-onset T1DM.

Variable	Total (*N* = 413)
Male (%)	205 (49.6)
Baseline Age (year)^a^	10.6 ± 4.5
Age at T1DM diagnosis (year)	8.4 ± 4.1
Overall duration of T1DM (month)	126.3 ± 53.4
Duration of follow-up (month)	97.0 ± 34.8
Baseline body mass index (kg/m^2^)	18.5 ± 2.9
Baseline HbA1c level (%)	10.0 ± 3.6
Mean lab results during the follow-up	
LDL cholesterol (mg/dl)	99.1 ± 27.9
>110 mg/dl, n (%)	127 (30.8)
Total cholesterol (mg/dl)	172.4 ± 31.2
>170 mg/dl, n (%)	199 (48.2)
Triglycerides (mg/dl)	74.6 ± 43.9
>90 mg/dl, n (%)	78 (18.9)
HDL cholesterol (mg/dl)	63.8 ± 12.3
<45 mg/dl, n (%)	14 (3.4)
Serum creatinine (mg/dl)	0.60 ± 0.21
>1.0 mg/dl, n (%)	3 (0.7)

^a^Age at registration in the Chang Gung Juvenile Diabetes Eye Study.

T1DM: Type 1 diabetes mellitus, HbA1c: hemoglobin A1c, LDL: low-density lipoprotein, HDL: high-density lipoprotein.

Continuous data were presented as mean ± standard deviation.

### Time-dependent analysis of risk factors

After using time-dependent Cox analysis of factors associated with DR, we identified four significant factors in both the multivariable model and the reduced model (Table 2). Male sex (hazard ratio [HR]: 0.4, 95% confidence interval [CI]: 0.19–0.85), onset of T1DM at age 5–14 years (HR: 6.38, 95% CI: 2.41–16.87), duration of T1DM (HR: 1.57, 95% CI: 1.41–1.75), and HbA1c level (HR: 1.56, 95% CI: 1.35–1.80) were independently associated with DR in the multivariable model. The reduced model was used to examine these four significantly associated factors, and similar results were obtained. Although correlations between NPDR, BMI, and lipid profiles (including LDL, cholesterol, triglyceride, and serum creatinine) were observed in the univariate analysis (data not shown), these relationships were no longer significant in the multivariable model. The reduced model had a superior discriminatory performance with a C-statistic of 0.923. We performed an internal validation using 200 bootstrap samples and found that the bootstrap-corrected C-statistic was 0.918. This trivial difference between the optimistic and bootstrap-corrected estimates suggested good generalisability of the model.

**Table 2:** Time-dependent Cox proportional hazard model analysis for non-proliferative diabetic retinopathy associated with juvenile-onset T1DM.

Variable	Multivariable model			Reduced model		
	B	HR (95% CI)	*P* value	B	HR (95% CI)	*P* value
Male	−0.921	0.40 (0.19, 0.85)	0.016	−0.672	0.51 (0.26, 0.99)	0.047
Onset of T1DM at age 5–14 yrs	1.852	6.38 (2.41, 16.87)	<0.001	1.701	5.48 (2.25, 13.38)	<0.001
Duration of T1DM (yr)^a^	0.451	1.57 (1.41, 1.75)	<0.001	0.439	1.55 (1.40, 1.72)	<0.001
Body mass index (kg/m^2^)	−0.049	0.95 (0.85, 1.07)	0.402			
HbA1c level (%)^a^	0.445	1.56 (1.35, 1.80)	<0.001	0.454	1.58 (1.39, 1.79)	<0.001
Annual mean of lab results						
LDL cholesterol (mg/dl)^b^	0.007	1.01 (1.00, 1.02)	0.139			
Triglycerides (mg/dl)^b^	0.010	1.01 (0.98, 1.04)	0.527			
HDL cholesterol (mg/dl)^b^	−0.010	0.99 (0.97, 1.01)	0.349			
Serum creatinine (mg/dl)^b^	0.851	2.34 (0.78, 7.02)	0.129			

T1DM: Type 1 diabetes mellitus, B: regression coefficient, CI: confidence interval, HR: hazard ratio, yr: year, HbA1c: hemoglobin A1c, LDL: low-density lipoprotein, HDL: high-density lipoprotein.

^a^Time-dependent covariate at each HbA1c exam.

^b^Time-dependent covariate using the mean value during a year of exam.

### Nomogram of NPDR risk

**Figure 2:**
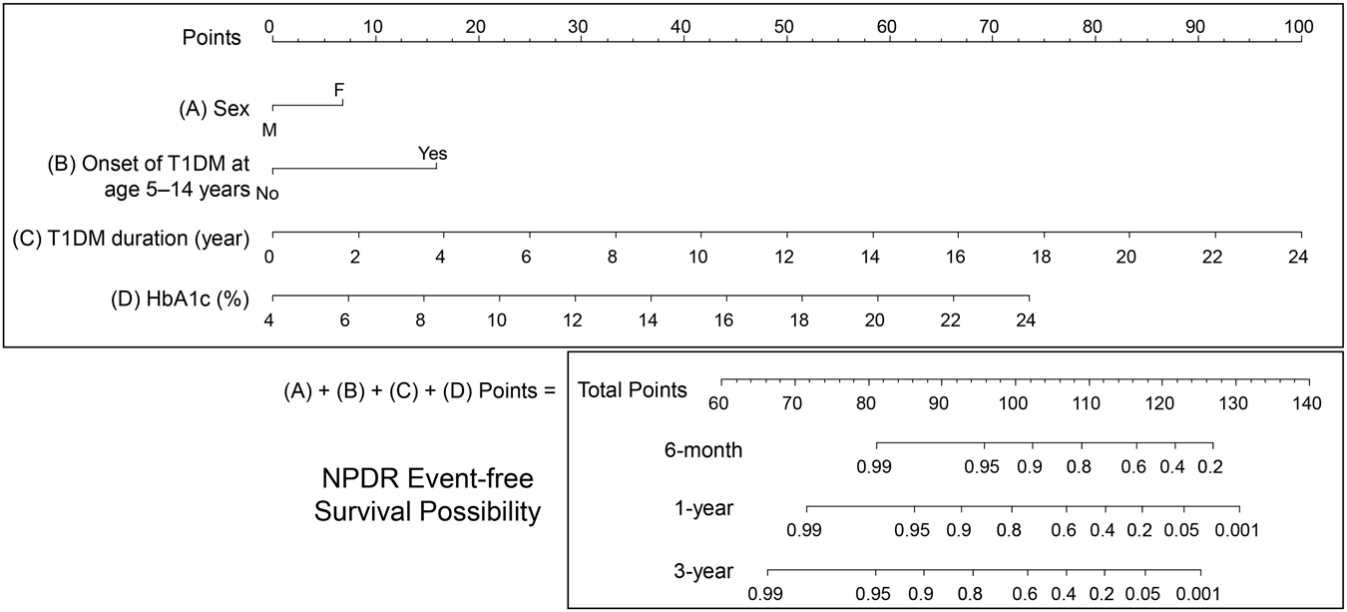
Prediction model for non-proliferative diabetic retinopathy. T1DM: type 1 diabetes mellitus, yrs: years, HbA1c: hemoglobin A1c, NPDR: non-proliferative diabetic retinopathy.

Based on the results of the time-dependent Cox analysis, a nomogram for NPDR risk prediction was built and is shown in Fig. 2. This formula offers a rapid way to interpret the DR risk predicted by the Cox model. A numeric scale was generated that allocated each significant covariate: ie, sex, age at T1DM onset, T1DM duration, and HbA1c level, a number of points according to the weight of its effect. With respect to the categorical variables, female sex scored 7 points, and the onset of T1DM at 5–14 years of age scored 16 points. For the continuous covariates, each year with T1DM increased the score by approximately 4.2 points, and 1% elevation of HbA1c level increased it by approximately 3.7 points. The total score derived from all the covariates indicated the probabilities of being DR-free in 6 months, 1 year, and 3 years. Using this figure, the risks of DR development could be estimated easily for an individual patient during clinical follow-up. For example, a 25-year-old woman who had T1DM for 17 years had a routine clinic follow-up, at which time her HbA1c was reported as 11%. She scored 7 points for female sex, 16 points for onset age, 70 points for disease duration, and 26 points for HbA1c, giving a total of 119 points. This score indicated probabilities of 0.5, 0.1, and <0.05 for being DR-free in 6 months, 1 year, and 3 years, respectively. Therefore, at that visit, the physician should encourage the patient to maintain better glycemic control or she would most likely develop NPDR within 3 years.

### Age and HbA1c

**Figure 3:**
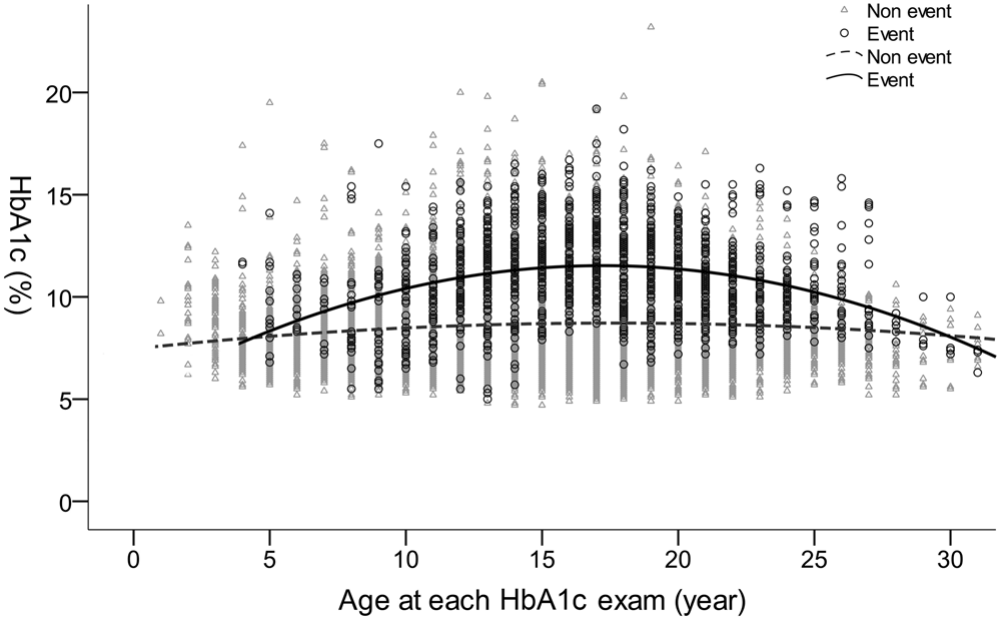
Correlation between age and HbA1c stratified by the presence of non-proliferative diabetic retinopathy (circles) and the absence of diabetic retinopathy (triangles). Mean of the HbA1c in the presence of non-proliferative diabetic retinopathy and the absence of diabetic retinopathy were presented as solid line and dashed line, respectively. HbA1c: hemoglobin A1c.

The correlation between age and HbA1c level in patients with and without NPDR is shown in Fig. 3. Each dot represents an individual record, and the two lines are the regression lines for HbA1c against age in the NPDR and non-DR groups. The HbA1c level in the NPDR group peaked at 17 years, whereas the level stayed steady in the non-DR group throughout the whole period.

## Discussion

To our knowledge, this is the first study that has used a nomogram to predict the risk of NPDR development in patients with juvenile-onset T1DM. This study developed a nomogram for use by clinicians to easily evaluate the risk of NPDR for individual T1DM patients. In the current study, 10.4% of patients (43/413) developed NPDR during a mean follow-up of 97 months. We identified four factors that were independently associated with NPDR development: sex, onset of T1DM at 5–14 years of age, duration of T1DM, and HbA1c level.

Since the Wisconsin Epidemiologic Study of Diabetic Retinopathy identified in 1984 that the duration of diabetes mellitus (DM) and serum HbA1c level were the most significant risk factors for NPDR development^17^, additional factors have been reported to be associated with DR in patients with juvenile-onset T1DM. Puberty and age at onset of T1DM, especially at 5–14 years of age, were reported as risks for retinopathy^18–20^. Other well-accepted risks include higher blood pressure^21,22^, greater HbA1c variability^23^, concomitant metabolic syndrome^24,25^, elevated lipid profile^15,19,26^, and familial factors^20^. Although progression and the advanced stages of DR ae more likely in males^19,22^, earlier development of DR in female patients with T1DM was reported in some studies, and earlier puberty in females was believed to play a role in this difference^20,27^.

Nomograms have been used to assess the probability of death in cancer patients. One study has demonstrated that a nomogram can help predict DR development in patients with type 2 diabetes mellitus^28^. Using a nomogram to predict the development of NPDR may assist the physician to evaluate the risk and encourage the patient’s glycemic control. In our study, we proposed a stratification of different NPDR risk factors in patients with juvenile-onset T1DM. We identified four factors independently associated with NPDR: sex, T1DM onset at 5–14 years of age, duration of T1DM, and HbA1c level. In our study, male sex was a protective factor, while onset of T1DM between 5–14 years of age carried the highest risk of DR development. In contrast, lipid profiles and BMI failed to show significance in the multivariate analysis. The stratification of these factors also allows the comparison of each type of risk. For example, a 1% increase of HbA1c may carry a similar risk to a 1-year increase in duration of T1DM, and a patient with T1DM onset at 5–14 years old may carry a risk comparable with four additional years duration of T1DM in a patient with T1DM onset at <5 years old or >14 years old.

With respect to the correlation between age and HbA1c, a higher HbA1c level was identified in the NPDR group, in which the increase in HbA1c peaked at about age 17, when the reproductive hormones have reached the highest level^29^. This finding implies that puberty may also play a role in HbA1c control in T1DM, and may consequently affect the development of NPDR. The reasons for this may include increased reluctance to comply with diabetes self-control, smoking, and a higher achievable glucose level at a young age^19,20,29,30^. Hence, intensive education about glycemic control and disease during adolescence may decrease the risks of NPDR in patients with T1DM.

Although lipid levels and BMI failed to show an independent association with NPDR development in the multivariable analysis, these factors were significantly associated with development of NPDR in the univariate analysis (HDL was not significant, data not shown). In our previous study, which followed patients for >10 years, we found that the serum triglyceride level was independently associated with development of NPDR^15^. In addition, many large-scale studies have reported dyslipidemia and BMI as risk factors^19,26,31^. There are several possible explanations for this discrepancy about whether dyslipidemia and BMI are independent risk factors of NPDR. First, it could take longer than the time frame of the current study for elevated serum lipids to cause progressive damage to retinal cells^32^. Second, dyslipidemia and BMI may be far less significant than other factors in the development of NPDR. Third, the effect of BMI might be mitigated when adjusting for other covariates, such as dyslipidemia and HbA1c level^33,34^.

We found that the mean HbA1c levels in the current study (8.8%) and our previous study (9.4%)^15^ were higher than those reported in Western populations (8.5% in Finland, 8.2% in the USA, and 7.5% in Sweden)^27,35,36^. This finding indicates poor glycemic control in juvenile-onset T1DM patients in Taiwan compared with those in Western countries. Although the mean HbA1c level is higher in T1DM patients in Taiwan, the prevalence of NPDR (10.4% in 97 months) is lower than those reported from Western countries (17% in 60 months in the US, 27.3% in 2 years in the UK)^37,38^. Genetic factors such as DR-associated alleles and environmental factors such as lifestyle or diet may contribute to this difference^2^.

Based on the current study, we developed a fast and physician-friendly nomogram that can not only evaluate the risk of NPDR development in patients with juvenile-onset T1DM but also provide a conversion of risk factors into disease-years. The conversion of risk into disease-years may also motivate patients to have better glycemic control. We analysed age as a time-dependent variable that was adjusted every time a patient visited our hospital for follow-up. The use of a time-dependent variable means that the prediction model is more reliable when patients are seen over different intervals during clinical follow-up.

There are some limitations to this study. First, there was no physical measurement of puberty such as the Tanner scale. Nevertheless, the results for age and HbA1c in the NPDR group (Fig. 3) suggested that there was a correlation between puberty and DR development. Second, we did not analyse the patients’ blood pressure annually, which has been reported as a risk factor^21,22^. However, blood pressure may show more variability, especially in agitated children, than the results of laboratory examinations. Third, this retrospective study was unable to assess the family history. In our clinical practice, mainly on weekdays, pediatric patients are sometimes came to the clinic themselves or brought to the clinic by relatives other than their parents, so information about the family history may not be reliable. Fourth, a three-field rather than seven-field fundus photography, which was used in the DCCT/EDIC study^39^, was performed, and our method may have underdiagnosed NPDR. Since the study was performed at the single medical centre in Taiwan, the number of study participant was relative small comparing to nation-wide studies. In addition, application of our results to other populations requires additional validation. Nevertheless, the study embraced a long follow-up duration (up to 97 months), so it still could provide a meaningful information to the issue. Finally, the study did not include functional tests such as multifocal electroretinograms, which have been reported to be able to predict development of DR in adult patients^40^. Ophthalmic functional studies including electroretinogram and visual field examination can also be considered standard for DR screening in institutes where this equipment is available. Application of these examinations in DR screening needs further investigation.

In conclusion, this is the first study using a nomogram to predict the risks of NPDR development in patients with T1DM. The risk factors for NPDR development in a Chinese population were also identified, and included female sex, onset of T1DM at 5–14 years of age, duration of T1DM, and the HbA1c level.
